# Diabetic Nephropathy-Associated Impaired Aortic Function Is Not Mediated by Mean Arterial Pressure and Its Determinants

**DOI:** 10.3390/jcm13247827

**Published:** 2024-12-21

**Authors:** Hon-Chun Hsu, Makabongwe S’kholiwe Mazibuko, Chanel Robinson, Noluntu Dlongolo, Angela Woodiwiss, Gloria Teckie, Grace Tade, Patrick Hector Dessein

**Affiliations:** 1 Cardiovascular Pathophysiology and Genomics Research Unit, School of Physiology, Faculty of Health Sciences, University of the Witwatersrand, Johannesburg 2193, South Africa; kengjulin@yahoo.com.au (H.-C.H.); 1353904@students.wits.ac.za (M.S.M.); chanelgr@gmail.com (C.R.); angela.woodiwiss@wits.ac.za (A.W.); grace.tade@hotmail.com (G.T.); 2 Nephrology Unit, Milpark Hospital, Johannesburg 2193, South Africa; 3 Rheumatology Unit, Rosebank Hospital, Johannesburg 2193, South Africa; dlongolonhlanhla@gmail.com; 4 Division of Nephrology, Department of Medicine, Chris Hani Baragwanath Hospital and Faculty of Health Sciences, University of Witwatersrand, Johannesburg 2193, South Africa; gteckie@hotmail.com; 5 Internal Medicine Department, University of the Witwatersrand, Johannesburg 2193, South Africa

**Keywords:** diabetic nephropathy, aortic function, mean arterial pressure, cardiac output, systemic vascular resistance

## Abstract

**Objective:** The study aimed to assess the potential impacts of mean arterial pressure (MAP) and its determinants (cardiac output and systemic vascular resistance) on diabetic nephropathy (DNP)-associated impaired aortic function. **Methods:** This multi-ethnic study included 115 chronic kidney disease (CKD) patients (67 non-dialysis and 48 dialysis). Six aortic function measures were evaluated by SpygmoCor. The stroke volume was determined by echocardiography. **Results:** Hypertensive nephropathy (HNP) (53.9%), DNP (32.2%), glomerulonephritis (19.1%), and HIV-associated nephropathy (7.8%) composed the major CKD etiologies. Concurrent HNP and DNP were present in 31.1% of the patients. Participants with compared with those without concurrent HNP and DNP experienced more frequent established cardiovascular disease (43.2% versus 14.9%, *p* = 0.01), a faster pulse wave velocity (*p* = 0.001), and smaller total arterial compliance as an indicator of proximal aortic stiffness (*p* = 0.03). DNP was associated with each aortic function measure (*p* < 0.001–0.02) independent of potential confounders and MAP, as well as its determinants. HNP was not related to aortic function (*p* > 0.05 for all relationships). MAP and its determinants did not mediate the potential impact of DNP on aortic function (−4.1–6.4% contribution). Covariates that were associated with impaired aortic function measures included MAP and its determinants (*p* < 0.001–0.01). **Conclusions:** Mean or distending arterial pressure and its determinants were associated with impaired aortic function in the overall CKD population. However, these hemodynamic factors did not mediate DNP-associated impaired aortic function. Our results suggest that blood pressure lowering can be anticipated to improve impaired aortic function in the overall CKD population but not when it is solely induced by DNP.

## 1. Introduction

More than 10% of the worldwide population experiences chronic kidney disease (CKD) [1]. Patients with CKD experience a markedly enhanced cardiovascular disease risk [2]. Cardiovascular disease mortality is increased up to 1000-fold in patients with kidney failure. Cardiovascular manifestations in CKD patients are atherosclerotic disease, heart failure, arrhythmias, and cardiac death. Most deaths in dialysis patients are attributable to sudden cardiac death [3].

Arterial stiffness [4] is a central pathophysiological mechanism in CKD-induced cardiovascular disease [5]. CKD-mediated oxidative stress, vascular inflammation, aberrant mineral and bone disease, sodium retention, and increased renin-angiotensin-aldosterone and sympathetic nervous system activity generate aortic stiffness. Carotid–femoral pulse wave velocity (CFPWV) currently composes the gold standard test for the evaluation of aortic stiffness [4]. Aortic stiffness increases the reflected Asor backward (Pb) and forward wave (Pf) amplitudes. Increased Pb and Pf amplitudes cause augmented pulsatile pressures, including central pulse pressure and thereby systolic blood pressure [6,7]. Enhanced Pb further translates into increased cardiac afterload [8] that mediates the development of uremic cardiomyopathy [9]. On the other hand, augmented Pf also increases pulsatile pressure transmission into low-resistance capillary beds of not only the brain but also the kidneys [10,11]. This causes microvascular damage that results in cognitive impairment and CKD progression, thereby closing the vicious circle between CKD and aortic stiffness [5].

A wide variety of illnesses can cause CKD. Globally, diabetes, hypertension, and glomerulonephritis are the most common causes of CKD [12]. However, there is a dearth of reported evidence on whether different CKD etiologies have disparate adverse effects on CVD risk. In this regard, a study by Ovrehus and colleagues [13] disclosed that among CKD patients who had undergone a kidney biopsy, overall survival was poorer, and progression to kidney failure was more rapid in those with compared with those without diabetic kidney disease. Importantly, also in the present context, HIV is extremely prevalent in South Africa, and HIV and/or its treatment are associated with CKD [14].

In a recent investigation among CKD patients from a large sub-Saharan low-income population, we found that hypertensive nephropathy (HNP) was by far the most common presumed cause of CKD [14]. This study unveiled a mutually independent relationship of both HNP and diabetic nephropathy (DNP) with pulsatile pressures including peripheral pulse pressure and systolic blood pressure. Most strikingly, in mediation analysis, steady state mean arterial pressure fully explained the potential impact of HNP on pulsatile pressures (103.9% to 115.7%). By contrast, mean arterial pressure did not account at all for the potential impact of DNP on pulsatile pressures (−2.0–7.5%). Importantly, in the present context, peripheral pulse pressure is reportedly a surrogate marker of arterial stiffness [7,15]. Mean arterial pressure causes arterial distension and thereby mediates organ perfusion. Mean arterial pressure is determined by volume load and systemic or peripheral vascular resistance and their interaction [16,17,18]. Accordingly, mean arterial pressure can be reduced by adequate volume overload management, as well as the use of vasodilators or antihypertensives [16,18]. Hence, we believe that our recent findings have implications in understanding the pathophysiology and the delineation of optimal CVD risk management in relation to CKD etiologies.

Herein, we assessed the potential impact of mean arterial pressure (MAP) and its determinants (cardiac output and systemic vascular resistance) on diabetic nephropathy (DNP)-associated impaired aortic function. Toward this purpose, we determined hemodynamic characteristics including six aortic function measures by applanation tonometry and cardiac parameters by echocardiography in CKD patients from a sub-Saharan middle- to high-income population.

## 2. Patients and Methods

### 2.1. Patients

This study was carried out in accordance with the Helsinki Declaration. The University of Witwatersrand Human (Medical) Research Committee approved the study (protocol number M15-08-43). Each patient provided written informed consent before participating. This investigation was multi-ethnic (black, 40.0%; Asian, 27.8%; white, 24.4%; mixed race, 7.8%) and enrolled 115 CKD patients (67 non-dialysis and 48 dialysis) at Milpark Hospital, Johannesburg, South Africa. Milpark Hospital is a private health care facility. In South Africa, persons that seek health care in private health care hospitals are those that typically have medical insurance and, accordingly, represent a middle- to high-income population. Exclusion criteria were a Chronic Kidney Disease Epidemiology Collaboration estimated glomerular filtration rate of ≥60 mL/min/1.73 m^2^, heart failure, active infection, and cancer.

### 2.2. Baseline Recorded Characteristics

The methods that were applied in the present study were reported previously [14,19,20]. Briefly, the baseline characteristics were demographic variables, anthropometric parameters, and major traditional and non-traditional or CKD-related cardiovascular risk factors. Established cardiovascular disease included ischemic heart disease (acute myocardial infarction, percutaneous transluminal coronary angioplasty, and coronary artery bypass graft), cerebrovascular disease (stroke and transient ischemic attack), and peripheral vascular disease. A cardiologist, neurologist, or vascular surgeon confirmed each of these diseases. All investigations were performed on a single day. Among dialysis patients, investigations were performed on a day prior to a hemodialysis session. Dialysis was performed thrice weekly using 4 h sessions. Most dialysis patients (72.9%) had an arteriovenous fistula (68.7%) or arteriovenous graft (4.2%). Each patient was in sinus rhythm at the time of the study.

We obtained etiological categories through patient record review. In each patient, the KDIGO Clinical Practice Guideline for the Evaluation and Management of Chronic Kidney Disease was routinely applied by H-CH during their first visit at the nephrology clinic [21]. In keeping with this guideline, we identified presumed hypertensive nephropathy (HNP) and diabetic nephropathy (DNP) based on clinical grounds without confirmation by kidney biopsies. Routine performance of kidney biopsies among patients with presumed HNP and DNP is considered inappropriate and unethical, especially when no clinical benefit is expected [13]. We recorded one or more diagnostic categories in each patient.

### 2.3. Hemodynamic Characteristics

Arterial function was determined using previously reported methods [22]. Brachial or peripheral blood pressure was recorded using the oscillometric SunTech device (SunTech Medical, 3827 S Miami Blvd, Suite 100, Morrisville, NC 27560, USA) [23]. We calculated the mean of ≥3 peripheral blood pressure measurements that were taken at least ≥30 s apart and subsequent to sitting quietly for at least ≥5 min.

The mean arterial blood pressure for the peripheral waveform was assessed electronically by the SphygmoCor device [20] (see below) and employing the following formula:

$$MP=\sum_{i=T_{0}}^{T_{f}}P_{i}/n$$

where T_0_ is the start of the waveform, T_F_ is the end of the waveform, P_i_ is pressure points, and n is the number of pressure points. We recorded the mean blood pressure during 8 consecutive heartbeats. This was performed only after the pulse waveform was consistent and had less than 5% variation in diastolic pressure and pulse height.

Aortic or central blood pressures and Pb and Pf amplitudes, as well as CFPWV, were assessed using a high-fidelity SPC-301 micromanometer (Millar instrument, Inc., Houston, Texas) that communicated with a computer utilizing SphygmoCor software, version 9.0 (AtCor Medical Pty. Ltd., West Ryde, New South Wales, Australia). Arterial waveforms at the radial (dominant arm) and carotid and femoral artery pulses were recorded subsequent to a 15 min rest in the supine position, for ten consecutive waveforms or heartbeats. A manual measurement by auscultation taken just before the arterial waveform recording was used to calibrate the pulse pressure. The peripheral pressure wave was converted into an aortic waveform using an extensively validated generalized transfer function that was incorporated in the SphygmoCor software [24,25]. When the systolic or diastolic variability of consecutive waveforms was more than 5% or the pulse wave signal amplitude was less than 80 mV, the results were discarded. We calculated the aortic pulse wave velocity as the distance in meters divided by the transit time in seconds. An ECG-derived R wave as a fiducial point was used to evaluate the time delay in the pulse waves between the carotid and femoral sites. The mean of 10 consecutive pulse waves represented the pulse transit time. The distance that the pulse wave traveled was represented by the difference between the distance from the femoral sampling site to the suprasternal notch and the distance from the carotid sampling site to the suprasternal notch. SphygmoCor software was used to determine the forward and reflected wave amplitude components of the aortic pressure waveform. SphygmoCor software separated the aortic waveform by using a modified triangular waveform. A single experienced observer (CR) who was unaware of the cardiovascular risk factor profiles of the patients performed all the measurements.

Echocardiography was carried out in line with the American Society of Echocardiography convention [26]. We used a Philips CX50 POC Compact CompactXtreme Ultrasound System (Philips Medical Systems (Pty) Ltd., 3000 Minuteman Road, Andover, MA 01810, USA) equipped with a 1.8–4.2 MHz probe that allowed for M-mode, 2-D, and tissue Doppler measurements. We positioned patients in the partial left decubitus position. The evaluated measures included the left ventricular geometry and systolic (lateral and midwall fractional shortening as longitudinal and circumferential myocardial contractility indices, respectively, and ejection fraction that represents the pump or chamber function) function.

The left ventricular dimensions comprised the left ventricular internal end diastolic and end systolic diameters and wall thickness (left ventricular septal and posterior wall thickness). These were assessed in the parasternal long-axis view by two-dimensional directed M-mode echocardiography. The left ventricular end diastolic volume was evaluated using the Teichholz method. The difference between the left ventricular end diastolic and systolic volumes as assessed by the Z-derived method constituted the stroke volume. Cardiac output was calculated as stroke volume × heart rate. We calculated systemic vascular resistance (SVR) as (mean arterial pressure − right atrial pressure)/cardiac output, assuming the right arterial pressure was 0 mmHg. The heart rate was assessed using the length of an averaged peripheral waveform captured during a 10 s period, using the following formula: 1000/the length of an averaged peripheral waveform recorded during a 10 s period × 60. We calculated the total arterial compliance (TAC) as stroke volume/aortic pulse pressure. TAC is a valuable marker of proximal aortic stiffness [20].

The observer who performed the arterial function assessments also performed the echocardiographic measurements (CR). The intra-observer echocardiographic measurement variability was assessed for left ventricular end-diastolic diameter, septal wall thickness, and posterior wall thickness. The intra-observer echocardiographic measurement variability was low with Pearson’s correlation coefficients of 0.92, 0.72, and 0.76 (*p* < 0.0001 for all, which confirmed the presence of consistently strong associations) and variances (mean % difference (SD)) of −0.41 (4.16), 0.45 (7.74), and 1.74 (6.08) for the left ventricular end-diastolic diameter, septal wall thickness, and posterior wall thickness, respectively.

### 2.4. Data Analysis

Continuous patient characteristics were expressed as mean (SD) when normally distributed and median (interquartile range (IQR)) when non-normally distributed. Categorical characteristics were given as proportions or percentages.

Eighty-four patients (73.0% of enrolled participants) had either only concurrent HNP and DNP, glomerular disease, HNP, or HIV-associated CKD. We compared the baseline characteristics and cardiovascular risk factors, as well as established and subclinical cardiovascular disease between the main etiological categories. The respective features were compared among the groups by employing one-way analysis of variance and the Kruskal–Wallis and Chi-squared tests for normally and non-normally distributed variables and categorical characteristics, respectively. Bonferroni correction was consistently applied for multiple comparisons. Age, sex, and black population origin-adjusted multivariate regression analysis was performed additionally when deemed appropriate.

We determined the mutually independent potential impacts of DNP and HNP on aortic function measures in multivariate regression models. In this analysis, we included age, female sex, black population origin, exercise status, hemoglobin concentrations, and erythropoietin stimulating agent use as potential confounders. These were previously identified in our setting [20]. Given that anthropometric measures and heart rate can also influence aortic function, body mass index (BMI) and heart rate were forced into the models. Subsequently, we entered the mean arterial pressure or its determinants including cardiac output and/or SVR or their interaction (cardiac output × SVR) in additional and consecutive models.

The contribution of mean arterial pressure and its most recognized determinant comprising cardiac output × SVR [20] to the potential impact of DNP and HNP on aortic function measures was determined in the confounder-adjusted product of the coefficient mediation analysis that considered hierarchical structures.

The impact of dialysis status on the relationships between DNP and aortic function was evaluated using the interaction term dialysis status × DNP in fully adjusted regression models. Stratified analysis by dialysis status was performed for CFPWV.

Data analysis was performed using Statistica 8.0 application package (version 14, TIBCO Software Inc., 3307 Hillview Avenue, Palo Alto, CA 94304, USA).

## 3. Results

### 3.1. Baseline Recorded Characteristics in CKD Patients

The baseline characteristics in all, non-dialysis, and dialysis patients that participated in the current investigation are provided in Supplementary Table S1. The mean (SD) age was 57.7 (14.0) years, and 37.4% were female. At the time of the study, hypertension, dyslipidemia, diabetes, and smoking were present in 90.4%, 79.8%, 34.8%, and 2.6% of the enrolled patients, respectively. The bulk of the study participants (76.5%) experienced uncontrolled systolic blood pressure despite the use of 2.2 (2.0) antihypertensive agents. Established cardiovascular disease was documented in 27.8% of patients.

Black patients were more often on dialysis, whereas white patients required dialysis less frequently. Dialysis patients exercised more frequently than those that were not dialyzed. Phosphate and parathyroid hormone levels were higher and hemoglobin concentrations were lower among dialysis patients than in their non-dialysis counterparts. Calcium channel blockers, diuretics, and erythrocyte-stimulating agents were employed more often by dialysis compared with non-dialysis patients. Sodium-glucose cotransporter-2 inhibitors were unavailable in South Africa at the study time (2016) and therefore not used in this cohort.

### 3.2. Etiological Categories in CKD Patients

**Figure 1:**
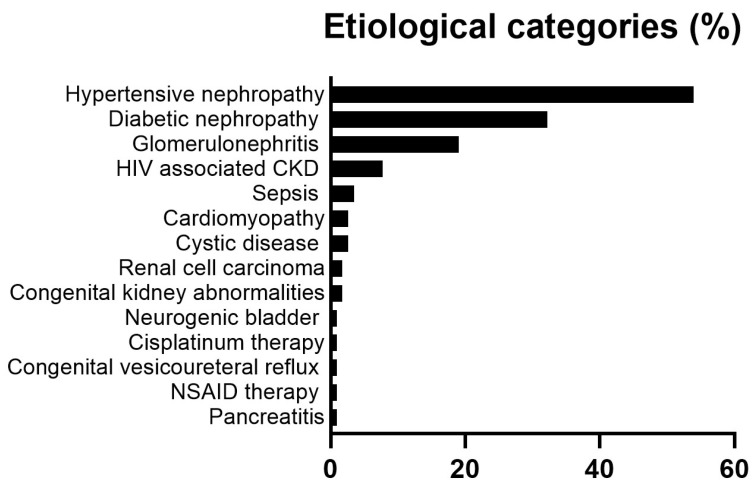
Etiological categories in 115 CKD patients. HIV, human immunodeficiency virus; CKD, chronic kidney disease; NSAID, non-steroidal anti-inflammatory agents.

The presumed etiological categories are given in Table 1 and further illustrated in Figure 1. The most prevalent etiological category comprised HNP (53.9%). This was followed by DNP (32.2%), glomerular disease (19.1%), and HIV-associated CKD (7.8%). Another 11 different etiologies were found in 0.9% to 3.5% of the study participants. The mean (SD) number of etiological categories was 1.3 (0.5). In age- and sex-adjusted logistic regression models, black population origin was associated with an increased prevalence of HNP (odds ratio (OR) (95% confidence interval (CI)) = 2.75 (1.20–6.31), *p* = 0.01) and DNP (OR (95% CI) = 3.70 (1.54–8.92), *p* = 0.004), and a reduced frequency of glomerular disease (OR (95% CI) = 0.13 (0.03–0.50), *p* = 0.003). In patients of both black and other population origins, HNP was more frequently recorded than DNP (65.2% versus 45.6% and 46.4% versus 23.2%, respectively).

### 3.3. Hemodynamic Characteristics in CKD Patients

The hemodynamic characteristics in all enrolled CKD patients are shown in Table S2. The mean peripheral systolic blood pressure (141 mmHg) was in the hypertensive range. The mean CFPWV was raised at 11.6 m/s (normal <10 mmHg) [27]. We recently reported a comparison of these features (except for peripheral pulse pressure, CFPWV, and Pb) between non-dialysis and dialysis CKD patients [20]. Whereas the mean (SD) central systolic blood pressure was larger in dialysis compared with non-dialysis participants (137 (19) mmHg versus 127 (17) mmHg, *p* value = 0.0007), none of the other hemodynamic characteristics differed among the two groups [20]. The mean (SD) peripheral pulse pressure, CFPWV, and Pb were also similar in dialysis and non-dialysis CKD patients (56 (17) mmHg versus 61 (21) mmHg, *p* = 0.3; 11.6 (3.6) m/s versus 12.0 (4.7) m/s, *p* = 0.4; and 23 (10) mmHg versus 21 (8) mmHg, *p* = 0.2, respectively).

### 3.4. Baseline Characteristics in the Main Isolated Etiological Categories Among CKD Patients

To ascertain whether etiological categories could impact cardiovascular risk factors and disease including aortic function, we singled out 84 CKD patients who had either only concurrent HNP and DNP (n = 37), glomerular disease (n = 22), HNP (n = 16), or HIV-associated CKD (n = 9). Only one patient with DNP did not have HNP. The baseline characteristics in CKD patients within the main isolated etiological categories are given in Table 2. In the current and following sections, only significant intergroup comparisons after Bonferroni correction at a *p* value of <0.05 are given.

**Figure 2:**
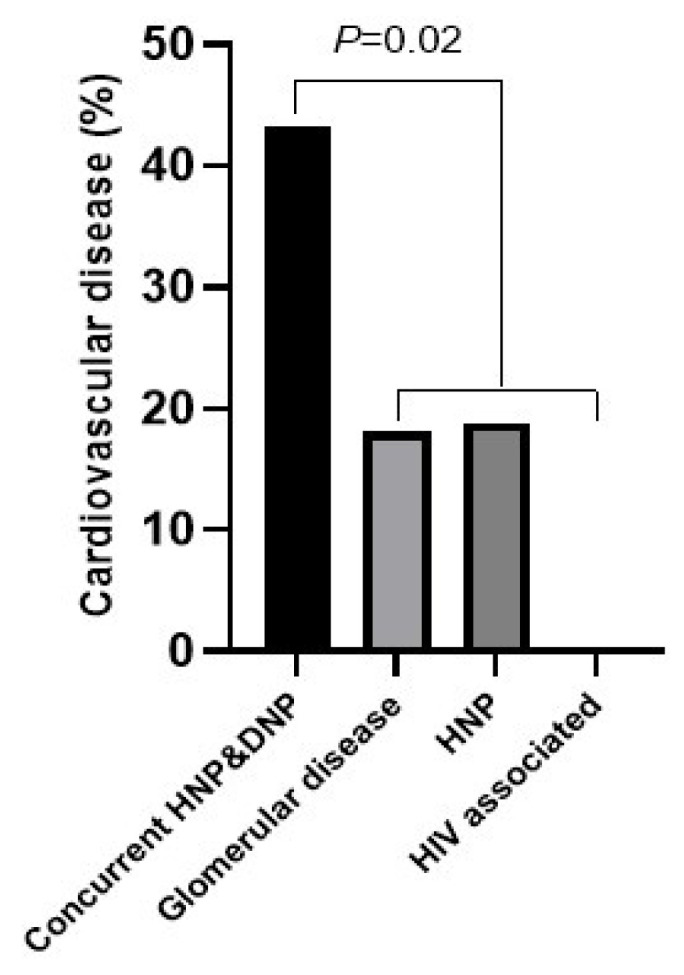
Significant differences in established cardiovascular disease among etiological categories. Established cardiovascular disease was more prevalent in patients with concurrent HNP and DNP than in those with glomerular disease, lone HNP, or HIV-associated chronic kidney disease. HNP, hypertensive nephropathy; DNP, diabetic nephropathy.

Patients with concurrent HNP and DNP were older than those with HIV-associated CKD (*p* < 0.003). Participants of black population origin had more frequently concurrent HNP and DNP, as well as HNP, compared with glomerular disease (*p* < 0.05 for both). Alcohol was consumed by only one patient who had HIV-associated CKD. The BMI (*p* = 0.03) and waist–height ratio (*p* = 0.007) were larger in patients with concurrent HNP and DNP than in those with glomerular disease. Only one patient with isolated HNP had developed diabetes by the time of the study. Patients with concurrent HNP and DNP experienced more frequently established cardiovascular disease than those with glomerular disease, HNP, and HIV-associated CKD (*p* < 0.05 for all). In a demographic characteristic-adjusted logistic regression model, concurrent HNP and DNP remained associated with established cardiovascular disease (OR (95% CI) = 3.45 (1.29–9.36), *p* = 0.01). Significant differences in established cardiovascular disease among etiological categories are further illustrated in Figure 2.

Further intergroup comparisons revealed that there were no differences in total cholesterol (*p* value = 0.2), low-density lipoprotein cholesterol (*p* value = 0.2), high-density lipoprotein cholesterol (*p* value = 0.2), and triglyceride concentrations (*p* value = 0.7).

### 3.5. Hemodynamic Characteristics in the Main Isolated Etiological Categories Among CKD Patients

**Figure 3:**
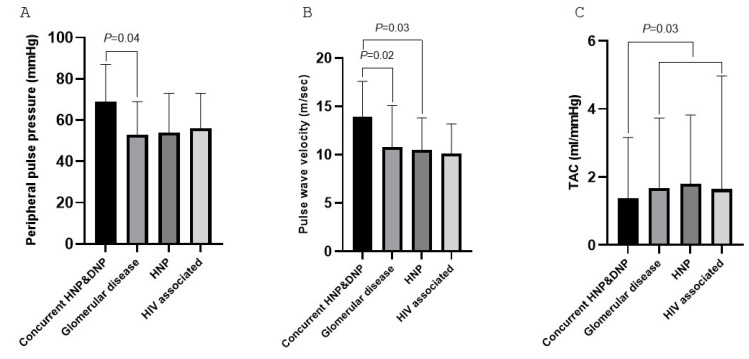
Significant differences in hemodynamic characteristics including peripheral pulse pressure (**A**), pulse wave velocity (**B**), and TAC (**C**) among the etiological categories. TAC was lower in patients with concurrent HNP and DNP compared with those with glomerular diseases, lone HNP, or HIV-associated chronic kidney disease. HNP, hypertensive nephropathy; DNP, diabetic nephropathy; TAC total arterial compliance.

The hemodynamic characteristics in CKD patients within the main isolated etiological categories are provided in Table 3. Peripheral pulse pressure was larger in patients with concurrent HNP and DNP than in those with glomerular disease (*p* = 0.04). CFPWV was larger in patients with concurrent HNP and DNP than in those with glomerular disease (*p* = 0.02) and HNP (*p* = 0.03). In an age-, sex-, and black population origin-adjusted linear regression model, concurrent HNP and DNP remained associated with CFPWV (model R^2^ = 0.165; β (SE) = 2.894 (0.861); *p* = 0.001). TAC was lower in patients with concurrent HNP and DNP (median (IQR) = 1.37 (1.02–1.79)) than in those within other categories (median (IQR) = 1.86 (1.37–2.09), *p* = 0.03) after adjustment for demographic characteristics. TAC was numerically larger in patients with HNP (median (IQR) = 1.80 (1.38–2.20)) compared with those within the other categories (median (IQR) = 1.57 (1.18–1.95)), but this difference did not reach significance (*p* = 0.1). Significant differences in hemodynamic characteristics among the etiological categories are further illustrated in Figure 3.

### 3.6. Confounder and Mutually Independent Potential Impacts of DNP and HNP on Aortic Function in CKD Patients

**Figure 4:**
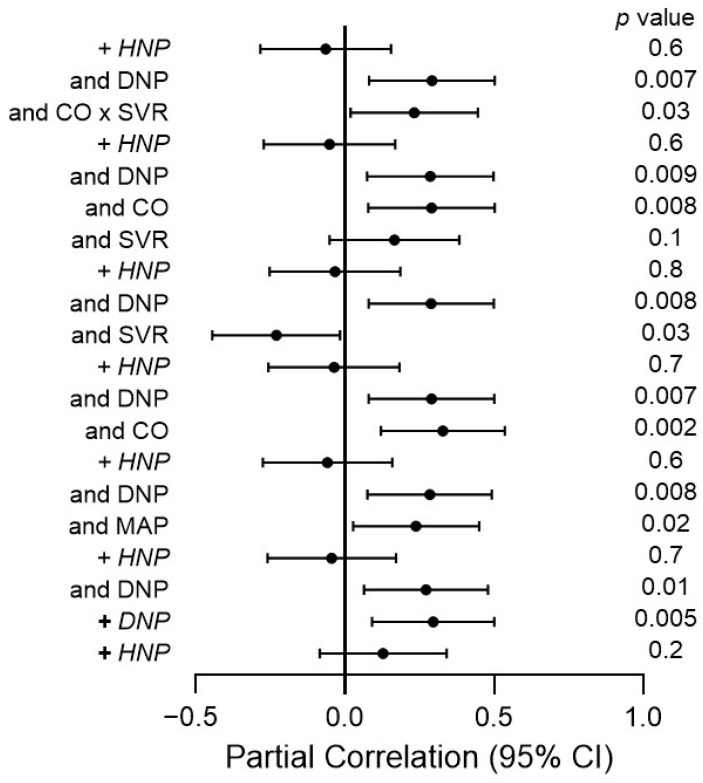
The partial correlation coefficients for the relationships in Table 7 (carotid–femoral pulse wave velocity). HNP, hypertensive nephropathy; DNP, diabetic nephropathy; CO, cardiac output; SVR, systemic vascular resistance.

The confounder and mutually independent potential impacts of DNP and HNP on aortic function measures are given in Table 4, Table 5, Table 6, Table 7 and Tables S3 and S4. The partial correlation coefficients for the relationships in Table 7 (CFPWV) are shown in Figure 4; those for the relationships in Table 4 (PPP), Table 5 (CPP), Table 6 (inverse of TAC), Tables S3 (Pb) and S4 (Pf) are given in the supplementary Figures S1 to S5, respectively. In the confounder-adjusted analysis, DNP was associated with peripheral pulse pressure (*p* = 0.001) (Table 4), central pulse pressure (*p* = 0.002) (Table 5), the inverse of TAC (*p* = 0.01) (Table 6), CFPWV (*p* = 0.005) (Table 7), and Pb (*p* = 0.007) (Table S3) and tended to be related to Pf (*p* = 0.06) (Table S4). HNP was not associated with any of the aortic function measures in the confounder-adjusted models (*p* = 0.2 to 0.8). Upon entering DNP and HNP into the same models, DNP was associated with all arterial function measures, whereas HNP was not related to aortic function. Upon adding mean arterial pressure to the models, DNP remained strongly associated with all aortic function measures. In the respective models, HNP was unrelated to the aortic function. Upon replacing the mean arterial pressure with one or both of its determinants comprising cardiac output and SVR or their interaction (cardiac output × SVR), DNP remained associated with all aortic function measures (*p* < 0.05 for all). In the respective models, HNP was unrelated to any of the aortic function measures. Covariates that were consistently associated with aortic function in regression models comprised the modifiable characteristics of mean arterial pressure and/or its determinants and/or their interaction.

In additional confounder-adjusted analyses, neither glomerular disease (β (SE) = −0.767, *p* value = 0.5) nor HIV-associated CKD (β (SE) = −1.667 (1.589), *p* value = 0.3) were significantly associated with CFPWV. Further adjustment for mean arterial pressure or its determinants or/and DNP did not alter these findings.

Current guidelines recommend targeting systolic blood pressure rather than pulse pressure in patients with CKD [28]. In this regard, systolic blood pressure is also an aortic function measure. Tables S5 and S6 show the relationships of DNP with peripheral and central systolic pressure, respectively. These associations did not differ from those with CFPWV in Table 7 (*p* = 0.1 to 0.4 for comparisons).

As given in Table S7, in a sensitivity analysis among the 83 CKD patients who did not have established cardiovascular disease, the relationships between DNP and CFPWV remained consistent.

As shown in Table 8, in the fully adjusted product of coefficient mediation analysis, mean arterial pressure or its determinant comprising cardiac output × SVR did not mediate (contribution = −4.4% to 6.4%) any of the potential effects of DNP on aortic function measures.

In fully adjusted models, the dialysis status did not influence any of the relationships of DNP with aortic function measures (interaction *p* = 0.3 to 9.0). Accordingly, as shown in Table S8, the relationships of DNP with CFPWV did not differ in non-dialysis compared with dialysis patients.

Whereas HNP was associated with peripheral pulse pressure and systolic blood pressure in our recently reported study [14], this was not the case in the current investigation. In addition to differences in socioeconomic status (low versus middle or high income), other potential explanations for this disparity among these two sub-Saharan African populations would comprise differences in proportions of patients of black population origin, potential confounding variables that were adjusted for, and entry criteria in the previous study (reduced EGFR or/and proteinuria) compared with only reduced EGFR in the present investigation. In this regard, black population origin did not impact the association of HNP with peripheral pulse and systolic blood pressure and CFPWV (interaction *p* = 0.9 for each) in the present study. Also, upon adjusting for the same potential confounding variables as in our previously reported investigation, which included age, female sex, black population origin, and DNP only, HNP remained unrelated to peripheral pulse pressure (β = −3.935 (4.216), *p* = 0.3), peripheral systolic blood pressure (β = −1.447 (4.845), *p* = 0.8), and CFPWV (β = −0.268 (0.998), *p* = 0.8). Finally, in a post hoc sensitivity analysis among patients with a reduced EGFR only in our previous study (n = 571) (14), HNP remained associated with peripheral pulse pressure (β = 4.420 (1.904), *p* = 0.02) and systolic blood pressure (β = 10.826 (2.731), *p* < 0.001).

## 4. Discussion

This study examined the disparate potential impacts of presumed etiological CKD categories on comprehensively evaluated aortic function. The main novel findings that emanated consecutively from our analysis were sevenfold. First, the most prevalent presumed cause of CKD was HNP (53.9%), and this was followed by DNP (32.2%), glomerular disease (19.1%), and HIV-associated CKD (7.8%). Second, patients with concurrent HNP and DNP experienced a markedly increased frequency of established cardiovascular disease when compared with those with lone HNP, glomerular disease, or HIV-associated CKD; concurrent HNP and DNP was associated with established cardiovascular disease independent of demographic characteristics (*p* = 0.01). Third, patients with concurrent HNP and DNP had a larger CFPWV (*p* = 0.001), as well as a smaller TAC (*p* = 0.03). Fourth, independent of confounders and HNP, as well as mean arterial pressure or its determinants, DNP was associated with impairment in each of the aortic function measures; by contrast, HNP was not independently related to aortic function. Fifth, mean arterial pressure or its most recognized determinant comprising cardiac output × SVR [21] did not mediate any of the potential adverse effects of DNP on aortic function (−4.4% to 6.4% contribution). Sixth, modifiable covariates that were consistently associated with aortic function measures in our analysis included mean arterial pressure or/and its determinants or/and their interaction. Seventh, the associations of DNP and HNP with CFPWV did not differ in non-dialysis compared with dialysis CKD patients. Taken together, in the current middle- to high-income South African patient cohort, HNP was the most frequently encountered presumed cause of CKD, and cardiovascular disease risk differed markedly by etiological CKD category. Equally if not more important, mean arterial pressure or its determinants did not mediate the potential adverse impact of DNP on aortic function.

The most striking findings in the present study were those concerning the potential impact of DNP on aortic function. As in our previous study [14], DNP was consistently associated with peripheral pulse pressure and systolic blood pressure independent of potential confounders and mediators. Our current findings further showed that this was invariably parallelled by relationships of DNP with other aortic function measures including the gold standard of CFPWV. This confirmed that peripheral pulse pressure could be useful in the routine evaluation of aortic function in patients with CKD. More importantly, whereas mean arterial pressure and its determinants were strongly associated with impaired aortic function in our regression models, these hemodynamic features did not mediate any of the DNP-associated impaired aortic function. We believe that this is the most innovative finding in the present study. As alluded to previously by us [20], mean arterial pressure can be modified by reducing volume overload or/and SVR through fluid intake restriction, diuretic therapy, dialysis, and antihypertensive agents or vasodilators. All in all, our results suggest that DNP-associated impaired aortic function is not attributable to mean or distending arterial pressure-induced vascular stretching and therefore represents intrinsic and hence likely irreversible or currently untreatable, as well as more severe, aortic stiffening. Future longitudinal studies are needed to determine whether our findings account for the larger mortality and more rapid CKD progression in patients with DNP compared with those with other nephropathies [13].

In contrast to our previous study [14], HNP was not associated with peripheral pulsatile pressures prior to adjusting for mean arterial pressure. This was also parallelled by a consistent absence of relationships of HNP with other aortic function measures. Intriguingly, this disparity between our previous [14] and present study could not be attributed to differences in the study entry criteria, the proportion of patients with a black population origin, confounders that were adjusted for, or dialysis status. Hence, how socioeconomic status can impact the potential effects of HNP on aortic function remains uncertain. In this regard, HNP-associated impaired aortic function that is potentially still reversible by reducing mean arterial pressure and therefore would be expected to precede intrinsic aortic stiffness may represent an early stage or more benign cardiovascular risk profile. Under such conditions, our results may represent a survival or selection bias as patients with advanced or long-standing aortic function impairment may have died prior to enrollment into our previous study. In line with this hypothesis, prevalent established CVD is also distinctly less often identified in sub-Saharan CKD patients from low-income compared with middle- or high-income populations (5.6% versus 27.8% in the present study) [14]. Noticeably, in the present context, low income is anticipated to enhance not only aortic stiffness [29] but also CKD progression [30]. Our current findings reinforce the previously recommended urgent need for further investigation into this issue [14].

The disparate potential impacts of HNP versus DNP on aortic function were pronounced and consistent in this study. In this regard, particularly patients with diabetes, as well as those with CKD, are distinctly vulnerable to developing arterial media calcification, which is expected to result in intrinsic aortic stiffness [2,31,32]. Whether aortic media calcification explains why DNP patients face more impaired aortic function than those with other nephropathies should be addressed in future studies.

The development of hypertension in patients with diabetes can increase the risk of CKD and its progression, as well as CVD incidence and mortality [33,34]. In the present investigation, patients with concurrent HNP and DNP had more frequent established CVD, faster CFPWV, and reduced TAC compared with the other study participants. These results confirm that different etiological CKD categories indeed have disparate effects on CVD risk. Patients with concurrent HNP and DNP experienced a particularly large cardiovascular risk burden including increased CFPWV and Pb, as well as established cardiovascular disease. Therefore, patients with concurrent HNP and DNP should be considered at exceptionally high risk of cardiovascular disease. As only one patient with DNP did not have HNP, we could not compare cardiovascular risk between those with concurrent HNP and DNP and lone DNP.

Whereas the inverse of TAC is a measure of proximal aortic stiffness [20], CFPWV does not measure stiffness in the ascending aorta [27]. We noted that in all study participants, mean arterial pressure and its most recognized determinant being cardiac output × SVR [20] were associated with each aortic function measure except for the inverse of TAC. Cardiac output and SVR were both associated with the inverse of TAC independent of confounders and one another. However, in addition to mean arterial pressure, the interaction between these two hemodynamic characteristics was also not independently related to the inverse of TAC. This suggests that mean arterial pressure can mediate descending aortic and iliac and femoral artery stiffness but not proximal aortic stiffness in patients with CKD. Interestingly, in this regard, Mitchell and colleagues previously reported that mean arterial pressure explained essential hypertension-related increased CFPWV but not proximal aortic stiffness [15]. Our findings together with previously reported data also indicate that volume control and blood pressure lowering can be anticipated to improve impaired aortic function in the overall CKD population.

In Europe, North and Latin America, and Asia [13,35,36,37], the most frequent cause of CKD is diabetes, which is followed by hypertension. The findings that were derived in our previous study [14], as well as our current results, indicate that HNP is the most common presumed cause of CKD in sub-Saharan Africa. This is followed by DNP. We previously speculated that this was because persons with a black African population origin are particularly prone to developing hypertension that is also often severe and resistant to treatment [14]. However, in the present study, HNP was more often encountered than DNP even among CKD patients that were not of black population origin. Our data indicate the need for rigorous prevention and management of both HNP and DNP in sub-Saharan Africans.

This investigation adjusted comprehensively for potential confounders in multivariate regression models that had a wide range of aortic function measures as dependent variables. The results were consistent across the different models. This study also has limitations. First, given our cross-sectional study design, the evaluation of cause–effect relationships cannot be performed. Second, we used the inverse of TAC as a measure of proximal aortic stiffness. The most recommended parameter for proximal aortic stiffness is Z_c_ [15]. In this regard, the inverse of TAC and characteristic impedance are strongly correlated (*R* = 0.83) [38]. Intrinsic proximal aortic stiffness due to the replacement of collagen with elastin and arterial media calcification explains ~50% of TAC [38,39]. Also, we recently documented in a population study that TAC was equally strongly related to pulsatile pressures as was characteristic impedance [20]. Herein, together with potential confounders, DNP and mean arterial pressure or its determinants accounted for up to 70% of the variation in aortic function measures. The primary aim of the present study was to assess the impacts of MAP and its determinants on DNP-associated impaired aortic function. We confirmed the previously reported hypothesis [14] that MAP and its determinants do not mediate the impact of DNP on aortic function. Nevertheless, the model R^2^ for the comprehensive models in Table 4, Table 5, Table 6, Table 7, Tables S3 and S4 was mostly below 0.700. This indicates the need for future preferably longitudinal investigations aimed at identifying additional patient characteristics or cardiovascular risk factors, other than those included in the present study, that may contribute to CKD-associated impaired aortic function. Third, although none of the enrolled participants were considered to have heart failure, prevalent heart failure with preserved ejection fraction is often undiagnosed in hemodialysis patients [40]. Fourth, we did not perform a power analysis prior to performing the present study. However, as currently recommended under the respective circumstances [41], we provided effect sizes (partial correlation coefficients) and their 95% confidence levels in conjunction with *p* values (see Figure 3 and Supplementary Figures S1–S5). Last, future studies should address the potential molecular mechanisms that could be involved in DNP-induced impaired aortic function. In this regard, vasopressin production is increased in diabetic kidney disease where it activates intra-renal renin–angiotensin–aldosterone system activation [42]. Vasopressin was also shown to suppress DNA synthesis in rat aortic smooth muscle cells [43]. We are planning to address the potential molecular mechanisms that can account for DNP-associated impaired aortic function in an ongoing study.

## 5. Conclusions

Mean or distending arterial pressure and its determinants are associated with impaired aortic function in the overall CKD population. However, these hemodynamic factors do not mediate DNP-associated impaired aortic function. Our results suggest that blood pressure lowering can be anticipated to improve impaired aortic function in the overall CKD population but not when it is solely induced by DNP.

## Figures and Tables

**Table 1:** Etiological categories in CKD patients.

Etiological Categories	CKD Patients (n = 115)
Hypertensive nephropathy	62 (53.9)
Diabetic nephropathy	37 (32.2)
Glomerular disease	22 (19.1)
HIV-associated CKD	9 (7.8)
Sepsis ^a^	4 (3.5)
Cardiomyopathy ^b^	3 (2.6)
Cystic disease	3 (2.6)
Renal cell carcinoma	2 (1.7)
Renal agenesis	1 (0.9)
Neurogenic bladder	1 (0.9)
Cisplatinum therapy	1 (0.9)
Congenital vesicoureteral reflux	1 (0.9)
NSAID therapy	1 (0.9)
Pancreatitis	1 (0.9)
Etiological categories	1.3 (0.5)

Data are expressed as number and proportions or mean (SD). CKD, chronic kidney disease; HIV, human immunodeficiency virus; NSAID, non-steroidal inflammatory agents. ^a^ These patients had experienced septic shock complicated by acute kidney injury that did not recover. ^b^ These patients had either ischemic or idiopathic cardiomyopathy complicated by reduced cardiac output.

**Table 2:** Baseline characteristics in the isolated major etiological categories among 84 CKD patients.

Etiological Categories					
Characteristics	Concurrent HNP and DNP (n = 37)	Glomerular Disease (n = 22)	HNP (n = 16)	HIV Associated (n = 9)	Intergroup Comparison *p* Value
**Demographics**					
Age (years)	**61.2 (12.2)**	**52.9 (14.6)**	**55.0 (14.0)**	**43.8 (7.6)**	**0.003**
Female sex (%)	35.1	50.0	31.2	33.3	0.6
Black (%)	**54.1**	**13.6**	**50.0**	**100**	**<0.001**
Asian (%)	32.4	36.4	31.2	0.0	0.2
White (%)	**10.8**	**36.4**	**12.5**	**0.0**	**0.03**
Mixed (%)	2.7	13.6	6.3	0.0	0.3
CKD duration (years)	5.2 (4.1)	6.9 (4.0)	4.6 (5.2)	2.6 (1.9)	0.06
Dialysis (%)	48.6	31.8	56.2	77.7	0.1
**Lifestyle factors**					
Alcohol use (%)	**0.0**	**0.0**	**0.0**	**11.1**	**0.03**
Exercise (%)	32.4	50.0	31.3	55.6	0.4
**Anthropometry**					
BMI (kg/m^2^)	**29.3 (5.7)**	**25.2 (5.4)**	**28.3 (5.5)**	**25.1 3.0)**	**0.02**
Waist–hip ratio	0.99 (0.13)	0.92 (0.12)	0.98 (0.05)	0.96 (0.05)	0.1
Waist–height ratio	**0.63 (0.09)**	**0.55 (0.11)**	**0.60 (0.10)**	**0.53 (0.09)**	**0.003**
**Traditional CV RFs**					
Hypertension (%)	97.3	86.4	93.8	88.9	0.4
Uncontrolled SBP (%)	78.4	68.2	75.0	77.8	0.8
Smoking (%)	2.7	0.0	0.0	0.0	0.7
Dyslipidemia (%)	74.3	90.0	92.9	55.6	0.08
Diabetes (%)	**100**	**0.0**	**6.3**	**0.0**	**<0.001**
**Non-traditional CV RFs**					
Dialysis duration (%)	36.0 (9.8–48.0)	12.0 (3.0–36.0)	24.0 (24.0–36.0)	36 (12.0–48.0)	0.5
EGFR (ml/min/1.73 m^2^)	34 (24)	35 (18)	39 (16)	32 (3)	0.9
Phosphate (mmol/l)	1.4 (0.5)	1.2 (0.5)	1.3 (0.7)	1.3 (0.3)	0.5
PTH (pg/mL)	245 (89–437)	81 (63–601)	179 (54–617)	275 (163–685)	0.4
Hemoglobin (g/dl)	12.0 (2.6)	12.3 (2.7)	10.9 (2.0)	10.4 (2.6)	0.2
**Treatment**					
Antihypertensive agent use (%)	97.3	86.4	93.8	88.9	0.4
Antihypertensives (n)	2.5 (1.2)	1.8 (1.2)	2.4 (1.1)	2.2 (1.3)	0.2
ACEI/ARB use (%)	72.2	81.8	93.8	88.9	0.3
Calcium channel blocker use (%)	55.6	36.4	43.8	55.6	0.5
Diuretic use (%)	**45.9**	**9.1**	**31.2**	**22.2**	**0.02**
Beta blocker use (%)	52.8	36.4	56.3	44.4	0.6
Alpha blocker use (%)	30.6	18.2	13.3	11.1	0.4
Statin use (%)	63.9	68.2	68.8	33.3	0.3
ESA use (%)	56.8	36.4	56.2	66.7	0.3
**Cardiovascular disease (%)**	**43.2**	**18.2**	**18.8**	**0.0**	**0.02**

Data are expressed as proportions, mean (SD), or median (interquartile range). Significant differences are given in bold. CKD, chronic kidney disease; HNP, hypertensive nephropathy; DNP, diabetic nephropathy; HIV, human immunodeficiency virus; EGFR, estimated glomerular filtration rate; PTH, parathyroid hormone; ACEI, angiotensin converting enzyme inhibitors; ARB, angiotensin receptor blockers; ESA, erythropoietin stimulating agents.

**Table 3:** Hemodynamic characteristics in the major isolated etiological categories among 84 CKD patients.

Etiological Categories					
Characteristics	Concurrent HNP and DNP (n = 37)	Glomerular Disease (n = 22)	HNP (n = 16)	HIV Associated (n = 9)	Intergroup Comparison *p* Value
Mean arterial pressure (mmHg)	102 (12)	102 (11)	105 (16)	104 (16)	0.8
Peripheral pulse pressure (mmHg)	**69 (18)**	**53 (16)**	**54 (19)**	**56 (17)**	**0.02**
Central pulse pressure (mmHg)	**52 (16)**	**41 (13)**	**41 (15)**	**42 (17)**	**0.03**
Peripheral systolic blood pressure (mmHg)	146 (21)	137 (20)	141 (21)	142 (26)	0.4
Central systolic blood pressure (mmHg)	135 (19)	131 (17)	124 (21)	135 (14)	0.3
TAC (ml/mmHg)	1.37 (1.01–1.79)	1.67 (1.22–2.06)	1.80 (1.38–2.02)	1.64 (1.37–3.33)	0.06
Pulse wave velocity (m/s)	**13.9 (3.7)**	**10.8 (4.3)**	**10.5 (3.3)**	**10.1 (3.1)**	**0.004**
Pb (mmHg)	**24.7 (8.0)**	**18.3 (7.4)**	**20.1 (7.3)**	**20 (8.8)**	**0.04**
Pf (mmHg)	34.5 (10.4)	29.2 (8.7)	33.3 (10.5)	34.6 (12.2)	0.4
Stroke volume (ml/beat)	69 (26)	65 (24)	70 (20)	83 (26)	0.3
Heart rate (beats/min)	77 (13)	73 (17)	79 (14)	77 (11)	0.7
Cardiac output (L/min)	5.4 (2.5)	4.6 (1.7)	5.5 (1.8)	6.2 (1.8)	0.3
SVR (mmHg/L per min)	21.0 (14.6–26.7)	24.5 (17.3–30.7)	21.0 (17.3–26.1)	16.5 (14.0–19.1)	0.4

Data are expressed as mean (SD) or median (interquartile range). Significant differences are given in bold. CKD, chronic kidney disease; HNP, hypertensive nephropathy; DNP, diabetic nephropathy; HIV, human immunodeficiency virus; TAC, total arterial compliance; Pb, reflected wave magnitude; Pf, forward wave magnitude; SVR, systemic vascular resistance.

**Table 4:** Confounder and mutually independent potential impacts of DNP and HNP on peripheral pulse pressure in CKD patients.

Characteristics	Cumulative R^2^	β (SE)	*p* Value	Std. β
Adjusted variables ^a^	0.164			
				
+HNP	0.168	2.739 (3.933)	0.5	0.071
				
+DNP	0.246	**12.918 (3.930)**	**0.001**	**0.311**
				
+HNP and	0.255	−4.886 (4.362)	0.3	−0.127
DNP		**15.555 (4.577)**	**<0.001**	**0.37**
				
+HNP and	0.388	−5.692 (3.980)	0.2	−0.148
DNP and		**15.784 (4.172)**	**<0.001**	**0.380**
MAP		**0.601 (0.131)**	**<0.001**	**0.386**
				
+HNP and	0.314	−3.641 (4.300)	0.4	−0.094
DNP and		**15.105 (4.496)**	**0.001**	**0.362**
Cardiac output		**2.872 (0.974)**	**0.004**	**0.294**
				
+HNP and	0.269	−3.797 (4.440)	0.4	−0.098
DNP and		**15.501 (4.641)**	**0.001**	**0.371**
Log SVR		−17.268 (11.188)	0.1	−0.154
				
+HNP and	0.363	−4.064 (4.169)	0.3	−0.105
DNP and		**13.978 (4.376)**	**0.002**	**0.335**
Cardiac output and		**8.396 (2.260)**	**<0 001**	**0.860**
Log SVR		**67.687 (25.167)**	**0.008**	**0.604**
				
+HNP and	0.383	−4.938 (3.918)	0.2	−0.128
DNP and		**15.486 (4.264)**	**<0.001**	**0.371**
Cardiac output × SVR		**0.601 (0.133)**	**<0.001**	**0.386**

Data were analyzed in multivariate regression models. DNP, diabetic nephropathy; HNP, hypertensive nephropathy; CKD, chronic kidney disease; β, regression coefficient; MAP, mean arterial pressure; SVR, systemic vascular resistance. ^a^ Variables that were adjusted for comprised age, female sex, black population origin, exercising status, hemoglobin concentration, erythropoietin stimulating agent use, body mass index, and heart rate.

**Table 5:** Confounder and mutually independent potential impacts of DNP and HNP on central pulse pressure in CKD patients.

Characteristics	Cumulative R^2^	β (SE)	*p* Value	Std. β
Adjusted variables ^a^	0.229			
				
+HNP	0.232	2.145 (3.204)	0.5	0.067
				
+DNP	0.302	**10.140 (3.192)**	**0.002**	**0.295**
				
+HNP and	0.311	−3.882 (3.560)	0.3	−0.121
DNP		**12.229 (3.720)**	**0.001**	**0.355**
				
+HNP and	0.540	−4.880 (2.927)	0.1	−0.153
DNP and		**12.672 (3.055)**	**<0.001**	**0.368**
MAP		**0.655 (0.096)**	**<0.001**	**0.513**
				
+HNP and	0.366	−3.124 (3.488)	0.4	−0.098
DNP and		**11.750 (3.631)**	**0.002**	**0.342**
Cardiac output		**2.482 (0.808)**	**0.003**	**0.311**
				
+HNP and	0.311	−3.198 (3.638)	0.4	−0.100
DNP and		**12.026 (3.785)**	**0.002**	**0.350**
Log SVR		−11.030 (9.321)	0.2	−0.120
				
+HNP and	0.457	−3.660 (3.251)	0.3	−0.115
DNP and		**10.755 (3.390)**	**0.002**	**0.313**
Cardiac output and		**8.621 (1.756)**	**<0.001**	**1.079**
Log SVR		**75.183 (19.433)**	**<0.001**	**0.820**
				
+HNP and	0.531	−4.333 (3.004)	0.1	−0.136
DNP and		**12.345 (3.124)**	**<0.001**	**0.359**
Cardiac output × SVR		**0.652 (0.098)**	**<0.001**	**0.514**

Data were analyzed in multivariate regression models. DNP, diabetic nephropathy; HNP, hypertensive nephropathy; CKD, chronic kidney disease; β, regression coefficient; MAP, mean arterial pressure; SVR, systemic vascular resistance. ^a^ Variables that were adjusted for comprised age, female sex, black population origin, exercising status, hemoglobin concentration, erythropoietin stimulating agent use, body mass index, and heart rate.

**Table 6:** Confounder and mutually independent potential impacts of DNP and HNP on the inverse of TAC in CKD patients.

Characteristics	Cumulative R^2^	β (SE)	*p* Value	Std. β
Adjusted variables ^a^	0.119			
				
+HNP	0.125	0.063 (0.076)	0.4	0.090
				
+DNP	0.175	**0.195 (0.077)**	**0.01**	**0.259**
				
+HNP and	0.178	−0.046 (0.087)	0.6	−0.065
DNP		0.220 (0.090)	**0.01**	**0.292**
				
+HNP and	0.185	−0.049 (0.087)	0.6	−0.071
DNP and		**0.221 (0.090)**	**0.01**	**0.294**
MAP		0.003 (0.003)	0.4	0.093
				
+HNP and	0.505	−0.057 (0.068)	0.4	−0.082
DNP and		**0.230 (0.070)**	**0.002**	**0.305**
Cardiac output		**−0.121 (0.016)**	**<0.001**	**−0.692**
				
+HNP and	0.676	−0.070 (0.055)	0.2	−0.101
DNP and		**0.209 (0.057)**	**<0.001**	**0.277**
Log SVR		**1.658 (0.140)**	**<0.001**	**0.824**
				
+HNP and	0.700	−0.075 (0.053)	0.1	−0.107
DNP and		**0.198 (0.055)**	**<0.001**	**0.262**
Cardiac output and		**0.077 (0.029)**	**0.009**	**0.437**
Log SVR		**2.423 (0.317)**	**<0.001**	**1.205**
				
+HNP and	0.185	−0.049 (0.087)	0.6	−0.071
DNP and		**0.221 (0.090)**	**0.01**	**0.294**
Cardiac output × SVR		0.003 (0.003)	0.4	0.093

Data were analyzed in multivariate regression models. DNP, diabetic nephropathy; HNP, hypertensive nephropathy; TAC, total arterial compliance; CKD, chronic kidney disease; β, regression coefficient; MAP, mean arterial pressure; SVR, systemic vascular resistance. ^a^ Variables that were adjusted for comprised age, female sex, black population origin, exercising status, hemoglobin concentration, erythropoietin stimulating agent use, body mass index, and heart rate.

**Table 7:** Confounder and mutually independent potential impacts of DNP. and HNP on CFPWV in CKD patients.

Characteristics	Cumulative R^2^	β (SE)	*p* Value	Std. β
Adjusted variables ^a^	0.167			
				
+HNP	0.181	1.065 (0.889)	0.2	0.130
				
+DNP	0.240	**2.572 (0.896)**	**0.005**	**0.290**
				
+HNP and	0.242	−0.424 (1.033)	0.7	−0.052
DNP		**2.817 (1.081)**	**0.01**	**0.318**
				
+HNP and	0.285	−0.541 (1.011)	0.6	−0.066
DNP and		**2.867 (1.056)**	**0.008**	**0.323**
MAP		**0.073 (0.032)**	**0.02**	**0.219**
				
+HNP and	0.326	−0.334 (1.041)	0.7	−0.040
DNP and		**2.883 (1.050)**	**0.007**	**0.325**
Cardiac output		**0.713 (0.226)**	**0.002**	**0.342**
				
+HNP and	0.285	−0.315 (1.047)	0.8	−0.038
DNP and		**2.962 (1.082)**	**0.008**	**0.333**
Log SVR		**−5.566 (2.604)**	**0.03**	**−0.231**
				
+HNP and	0.345	−0.469 (1.010)	0.6	−0.057
DNP and		**2.807 (1.043)**	**0.009**	**0.316**
Cardiac output and		**1.433 (0.526)**	**0.008**	**0.687**
Log SVR		8.882 (5.864)	0.1	0.369
				
+HNP and	0.285	−0.609 (1.043)	0.6	−0.073
DNP and		**2.983 (1.081)**	**0.007**	**0.336**
Cardiac output × SVR		**0.071 (0.033)**	**0.03**	**0.214**

Data were analyzed in multivariate regression models. DNP, diabetic nephropathy; HNP, hypertensive nephropathy; CFPWV, carotid–femoral pulse wave velocity; CKD, chronic kidney disease; β, regression coefficient; MAP, mean arterial pressure; SVR, systemic vascular resistance. ^a^ Variables that were adjusted for comprised age, female sex, black population origin, exercising status, hemoglobin concentration, erythropoietin stimulating agent use, body mass index, and heart rate.

**Table 8:** MAP^a^ and CO × SVR ^a^ as mediators of DNP on aortic function measures.

Aortic Function Measure	MAP as Mediator		CO × SVR as Mediator	
**PPP**	**Estimate (95% CI)**	**% Contribution**	**Estimate (95% CI)**	**% Contribution**
Direct effect	12.71 (5.58–19.85	98.5	12.84 (5.55–20.12)	98.1
Indirect effect	0.20 (−3.21–4.31)	1.5	0.25 (−3.26–4.38)	1.9
Total effect	12.91 (5.12–20.71)	100	13.09 (5.13–21.04)	100
**CPP**				
Direct effect	10.05 (4.80–15.29)	99.1	10.03 (4.68–15.39)	98.8
Indirect effect	0.09 (−3.71–4.43)	0.9	0.13 (−3.60–4.26)	1.2
Total effect	10.14 (3.80–16.48)	100	10.16 (3.70–16.62)	100
**Inverse of TAC**				
Direct effect	0.19 (0.04–0.35)	99.7	0.19 (0.04–0.35)	99.8
Indirect effect	0.00 (−0.02–0.03)	0.3	0.00 (−0.02–0.03)	0.2
Total effect	0.19 (0.04–0.35)	100	0.19 (0.04–0.35)	100
**CFPWV**				
Direct effect	2.55 (0.81–4.30)	99.3	2.63 (0.85–4.41)	98.5
Indirect effect	0.02 (−0.49–0.65)	0.7	0.04 (−0.45–0.65)	1.5
Total effect	2.57 (0.79–4.35)	100	2.67 (0.86–4.49)	100
**Pb**				
Direct effect	5.38 (2.22–8.54)	103	5.13 (1.87–8.38)	103.6
Indirect effect	−0.16 (−2.18–2.24)	−3	−0.18 (−2.26–2.12)	−3.6
Total effect	5.22 (1.49–8.94)	100	4.95 (1.11–8.78)	100
**Pf**				
Direct effect	4.71 (0.41–9.01)	104.1	4.84 (0.43–9.25)	104.4
Indirect effect	−0.18 (−2.53–2.45)	−4.1	−0.21 (−2.60–2.58)	−4.4
Total effect	4.53 (−0.31–9.37)	100	4.63 (−0.33–9.60)	100
**PSBP**				
Direct effect	8.48 (3.72–13.24)	94.7	8.56 (3.70–13.41)	93.6
Indirect effect	0.48 (−7.41–9.05)	5.3	0.58 (−7.72–9.26)	6.4
Total effect	8.96 (0.01–17.89)	100	9.14 (0.03–18.25)	100
**CSBP**				
Direct effect	5.65 (−1.91–13.21)	103.5	6.27 (−1.49–14.03)	103.7
Indirect effect	−0.18 (−4.76–4.98)	−3.5	−0.22 (−4.77–5.32)	−3.7
Total effect	5.47 (−3.13–14.05)	100	6.05 (−2.81–14.91)	100

The results were derived from product of coefficient mediation analysis. Adjustments were made for age, female sex, black population origin, exercise status, hemoglobin concentrations, erythropoietin stimulating agent use, and hypertensive nephropathy. MAP, mean arterial pressure; CO, cardiac output; SVR, systemic vascular resistance; DNP, diabetic nephropathy; PPP, peripheral pulse pressure; CPP, central pulse pressure; TAC, total arterial compliance; CFPWV, carotid–femoral pulse wave velocity; Pb, reflected wave amplitude; PSBP, peripheral systolic blood pressure; CSBP, central systolic blood pressure. ^a^ Mediation by these hemodynamic factors is represented by indirect effects.

## Data Availability

All relevant data are contained within the manuscript.

## Supplementary Materials

The following supporting information can be downloaded at https://www.mdpi.com/article/10.3390/jcm13247827/s1: Table S1. Baseline characteristics in CKD patients. Table S2. Hemodynamic characteristics in CKD patients. Table S3. Confounder and mutually independent potential impacts of DNP and HNP on Pb in CKD patients. Table S4. Confounder and mutually independent potential impacts of DNP and HNP on Pf in CKD patients. Table S5. Confounder and mutually independent potential impacts of DNP and HNP on peripheral systolic pressure in CKD patients. Table S6. Confounder and mutually independent potential impacts of DNP and HNP on central systolic pressure in CKD patients. Table S7. Confounder and mutually independent potential impacts of DNP and HNP on CFPWV in 83 CKD patients without CVD. Table S8. Confounder and mutually independent potential impacts of DNP and HNP on CFPWV non-dialysis and dialysis CKD patients. Figure S1. Partial correlations for the models in Table 5 (peripheral pulse pressure). DNP, diabetic nephropathy; HNP, hypertensive nephropathy; MAP; mean arterial pressure; log, logarithmically transformed; SVR, systemic vascular resistance. Figure S2. Partial correlations for the models in Table 6 (central pulse pressure). DNP, diabetic nephropathy; HNP, hypertensive nephropathy; MAP; mean arterial pressure; log, logarithmically transformed; SVR, systemic vascular resistance. Figure S3. Partial correlations for the models in Table 7 (the inverse of total arterial compliance). DNP, diabetic nephropathy; HNP, hypertensive nephropathy; MAP; mean arterial pressure; log, logarithmically transformed; SVR, systemic vascular resistance. Figure S4. Partial correlations for the models in Table S3 (reflective wave amplitude). DNP, diabetic nephropathy; HNP, hypertensive nephropathy; MAP; mean arterial pressure; log, logarithmically transformed; SVR, systemic vascular resistance. Figure S5. Partial correlations for the models in Table S4 (forward wave amplitude). DNP, diabetic nephropathy; HNP, hypertensive nephropathy; MAP; mean arterial pressure; log, logarithmically transformed; SVR, systemic vascular resistance.
