# Supplementary material for: Diabetic Nephropathy-Associated Impaired Aortic Function Is Not Mediated by Mean Arterial Pressure and Its Determinants
Source: J Clin Med. 2024 Dec 21;13(24):7827. doi: 10.3390/jcm13247827 (PMC11727795; doi:10.3390/jcm13247827)
Supplement: Supplementary file 1 [file jcm-13-07827-s001.zip › jcm-3324504-supplementary.pdf]

Table S1. Baseline characteristics in CKD patients

Characteristics	All patients (n=115)	Non-dialysis patients (n=67)	Dialysis patients (n=48)	*p*-Value
**Demographics**				
Age (years)	57.7 (14.0)	59.0 (13.8)	59.0 (13.8)	0.7
Female sex (%)	37.4	31.3	45.8	0.1
Black (%)	40.0	**28.4**	**56.2**	**0.005**
Asian (%)	27.8	31.3	22.9	0.2
White (%)	24.4	**35.8**	**8.3**	**0.003**
Mixed (%)	7.8	4.5	12.5	0.1
CKD duration (years)	5.4 (4.5)	6.0 (5.2)	4.6 (3.3)	0.3
Dialysis (%)	41.7			
**Lifestyle factors**				
Alcohol use (%)	1.7	1.5	2.1	0.8
Exercise (%)	36.5	**25.4**	**52.1**	**0.003**
Anthropometry				
BMI (kg/m^2)	27.4	27.8 (5.3)	26.8 (5.6)	0.5
Waist-hip ratio	0.97 (0.11)	0.96 (0.11)	0.98 (0.09)	0.2
Waist-height ratio	0.59 (0.09)			
**Traditional cardiovascular RFs**				
Hypertension (%)	90.4	86.6	95.8	0.8
Uncontrolled SBP (%)	76.5	74.6	79.2	0.7
Smoking (%)	2.6	4.5	0.0	-
Dyslipidemia (%)	79.8	85.5	71.4	0.2
Diabetes (%)	34.8	31.3	39.6	0.6
**Non-traditional cardiovascular RFs**				
Dialysis duration (%)	24 (12-36)		24 (12-36)	
EGFR (ml/min/1.73 m^2)	35 (21)	35 (21)		
Phosphate (mmol/l)	1.3 (0.5)	**1.2 (0.5)**	**1.4 (0.6)**	**0.02**
PTH (pg/ml)	174 (69-507)	**83.0 (56.0-159.5)**	**502.8 (182.0-785.7)**	**<0.001**
Haemoglobin (g/dl)	12.0 (2.6)	**12.8 (2.8)**	**10.8 (1.7)**	**0.001**
**Treatment**				
Antihypertensive agent use (%)	90.4	86.6	95.8	0.3
Antihypertensives (n)	2.2 (2.0)	2.1 (1.3)	2.4 (1.1)	0.3
ACEI/ARB use (%)	80.4	80.3	80.4	1.0
Calcium channel blocker use (%)	43.4	**31.3**	**61.0**	**0.01**
Diuretic use (%)	34.2	36.4	31.2	0.3
Beta blocker use (%)	45.5	37.9	56.5	0.04
Alpha blocker use (%)	21.6	22.7	20.0	0.6
Statin use (%)	64.3	69.7	56.5	0.9
ESA use (%)	47.0	**16.4**	**89.6**	**<0.001**
**Cardiovascular disease (%)**	27.8	26.9	29.2	0.2

Data are expressed as mean (SD), median (interquartile range) or proportions and analysed in age, sex and black population origin adjusted regression models. Significant differences in non-dialysis versus dialysis patients are shown in bold. CKD, chronic kidney disease; RFs, risk factors; EGFR, estimated glomerular filtration rate; PTH, parathyroid hormone; ACEI, angiotensin converting enzyme inhibitors; ARB, angiotensin receptor blockers; ESA, erythropoietin stimulating agents.

Table S2. Haemodynamic characteristics in CKD patients

Characteristics	CKD patients (n=115)
Mean arterial pressure (mmHg)	102 (12)
Peripheral pulse pressure (mmHg)	58 (19)
Central pulse pressure (mmHg)	45 (16)
Peripheral systolic blood pressure (mmHg)	141 (21)
Central systolic blood pressure (mmHg)	130 (19)
TAC (ml/minHg)	1.61 (1.22-1.98)
Pulse wave velocity (m/sec)	11.6 (4.1)
Pb (mmHg)	21 (9)
Pf (mmHg)	32 (11)
Stroke volume (ml/beat)	70 (24)
Heart rate (beats/min)	75 (14)
Cardiac output (L/min)	5.2 (2.0)
SVR (mmHg/L per min)	20.1 (15.4-26.7)

Data are expressed as mean (SD) or median (interquartile range). CKD, chronic kidney disease; TAC, total arterial compliance; Pb, reflected wave magnitude; Pf, forward wave magnitude; SVR, systemic vascular resistance.

Table S3. Confounder and mutually independent potential impact of DNP and HNP on Pb in CKD patients

	Cumulative R^2	β (SE)	*p* Value	Std. β
Adjusted variables[a]	0.217			
+HNP	0.223	1.392 (1.864)	0.4	0.079
+DNP	0.284	**5.220 (1.873)**	**0.007**	**0.274**
+HNP and	0.290	-1.711 (2.109)	0.4	-0.096
DNP		**6.165 (2.209)**	**0.007**	**0.324**
+HNP and	0.499	-2.010 (1.783)	0.3	-0.113
DNP and		**6.492 (1.867)**	**<0.001**	**0.341**
MAP		**0.336 (0.058)**	**<0.001**	**0.484**
+HNP and	0.319	-1.522 (2.106)	0.5	-0.087
DNP and		**5.943 (2.206)**	**0.009**	**0.314**
Cardiac output		**1.185 (0.509)**	**0.02**	**0.251**
+HNP and	0.277	-1.497 (2.171)	0.5	-0.085
DNP and		**5.853 (2.275)**	**0.01**	**0.309**
Log SVR		-4.167 (5.915)	0.5	-0.077
+HNP and	0.413	-1.903 (1.970)	0.3	-0.108
DNP and		**5.706 (2.063)**	**0.007**	**0.301**
Cardiac output and		**4.777 (1.127)**	**<0.001**	**1.011**
Log SVR		**44.656 (12.709)**	**<0.001**	**0.825**
+HNP and	0.484	-1.810 (1.835)	0.3	-0.103
DNP and		**6.119 (1.922)**	**0.002**	**0.323**
Cardiac output x SVR		**0.333 (0.059)**	**<0.001**	**0.487**

Data were analysed in multivariate regression models. DNP, diabeticnephropathy; HNP, hypertensive nephropathy; Pb, reflected wave amplitude; CKD, chronic kidney disease; β, regression coefficient; PO, population origin; ESA, erythropoietin stimulating agents; MAP, mean arterial pressure; SVR, systemic vascular resistance.

[a]Variables that were adjusted for comprised age, female sex, black population origin, exercising status, haemoglobin concentration, erythropoietin stimulating agent use, body mass index and heart rate.

Table S4. Confounder and mutually independent potential impact of DNP and HNP on Pf in CKD patients

Characteristics	Cumulative R^2	β (SE)	*p* Value	Std. β
Adjusted variables[a]	0.144			
+HNP	0.144	0.540 (2.372)	0.8	0.025
+DNP	0.178	4.529 (2.435)	0.06	0.196
+HNP and	0.186	-2.409 (2.740)	0.4	-0.112
DNP		**5.859 (2.869)**	**0.04**	**0.254**
+HNP and	0.370	-2.750 (2.425)	0.3	-0.128
DNP and		**6.232 (2.540)**	**0.01**	**0.270**
MAP		**0.382 (0.079)**	**<0.001**	**0.454**
+HNP and	0.257	-1.808 (2.689)	0.5	-0.084
DNP and		**5.878 (2.817)**	**0.04**	**0.254**
Cardiac output		**1.831 (0.650)**	**0.006**	**0.317**
+HNP and	0.209	-1.713 (2.776)	0.5	-0.080
DNP and		**5.834 (2.909)**	**0.04**	**0.252**
Log SVR		-12.383 (7.562)	0.1	-0.187
+HNP and	0.297	-2.111 (2.636)	0.4	-0.098
DNP and		**5.690 (2.760)**	**0.04**	**0.246**
Cardiac output and		**4.694 (1.508)**	**0.003**	**0.813**
Log SVR		**35.598 (17.004)**	**0.04**	**0.538**
+HNP and	0.366	-2.142 (2.486)	0.4	-0.100
DNP and		**6.016 (2.604)**	**0.02**	**0.260**
Cardiac output x SVR		**0.380 (0.080)**	**<0.001**	**0.454**

Data were analysed in multivariate regression models. DNP, diabetic nephropathy; HNP, hypertensive nephropathy; Pf, forward wave amplitude; CKD, chronic kidney disease; β, regression coefficient; PO, population origin; ESA, erythropoietin stimulating agents; MAP, mean arterial pressure; SVR, systemic vascular resistance.

[a]Variables that were adjusted for comprised age, female sex, black population origin, exercising status, haemoglobin concentration, erythropoietin stimulating agent use, body mass index and heart rate.

Table S5. Confounder and mutually independent potential impact of DNP and HNP on peripheral systolic pressure in CKD patients

Characteristics	Cumulative R^2	β (SE)	*p* value	Std. β
Adjusted variables[a]	0.148			
+HNP	0.152	2.980 (4.368)	0.5	0.070
+DNP	0.181	**8.954 (4.506)**	**0.05**	**0.169**
+HNP and	0.182	-1.917 (5.029)	0.7	-0.045
DNP		9.989 (5.277)	0.06	0.218
+HNP and	0.775	-3.795 (2.653)	0.2	-0.090
DNP and		**10.523 (2.781)**	**<0.001**	**0.230**
MAP		**1.401 (0.087)**	**<0.001**	**0.818**
+HNP and	0.280	-0.752 (4.834)	0.9	-0.018
DNP and		9.636 (5.055)	0.06	0.210
Cardiac output		**4.016 (1.095)**	**<0.001**	**0.375**
+HNP and	0.185	-1.236 (5.147)	0.8	-0.029
DNP and		10.056 (5.380)	0.06	0.219
Log SVR		-11.520 (12.970)	0.4	-0.094
+HNP and	0.528	-1.798 (3.937)	0.6	-0.042
DNP and		6.851 (4.132)	0.1	0.149
Cardiac output and		**17.660 (2.134)**	**<0.001**	**1.649**
Log SVR		**167.166 (23.765)**	**<0.001**	**1.360**
+HNP and	0.772	-3.292 (2.719)	0.2	2.719
DNP and		**10.324 (2.842)**	**<0.001**	**2.842**
Cardiac output x SVR		**1.401 (0.089)**	**<0.001**	**0.820**

Data were analysed in multivariate regression models. DNP, diabetic nephropathy; HNP, hypertensive nephropathy; CKD, chronic kidney disease; β, regression coefficient; PO, population origin; ESA, erythropoietin stimulating agents; MAP, mean arterial pressure; SVR, systemic vascular resistance.

[a]Variables that were adjusted for comprised age, female sex, black population origin, exercising status, haemoglobin concentration, erythropoietin stimulating agent use, body mass index and heart rate.

Table S6. Confounder and mutually independent potential impact of DNP and HNP on central systolic pressure in CKD patients

Characteristics	Cumulative R^2	β (SE)	*p* value	Std. β
Adjusted variables[a]	0.256			
+HNP	0.259	-2.554 (4.025)	0.5	-0.067
+DNP	0.269	5.458 (4.321)	0.2	0.130
+HNP and	0.288	-6.699 (4.570)	0.1	-0.176
DNP		9.046 (4.940)	0.07	0.215
+HNP and	0.459	-6.658 (4.006)	0.1	-0.175
DNP and		**9.212 (4.330)**	**0.03**	**0.219**
MAP		**0.691 (0.135)**	**<0.001**	**0.219**
+HNP and	0.292	-6.725 (4.782)	0.2	-0.174
DNP and		9.600 (5.120)	0.06	0.224
Cardiac output		-0.196 (1.178)	0.9	-0.018
+HNP and	0.312	-7.159 (4.710)	0.1	-0.185
DNP and		9.829 (5.045)	0.05	0.230
Log SVR		19.388 (12.674)	0.1	0.160
+HNP and	0.391	-6.604 (4.461)	0.1	-0.171
DNP and		9.514 (4.776)	0.05	0.222
Cardiac output and		**8.639 (2.704)**	**0.002**	**0.812**
Log SVR		**105.583 (29.519)**	**<0.001**	**0.870**
+HNP and	0.467	-6.464 (4.136)	0.1	-0.167
DNP and		**9.683 (4.438)**	**0.03**	**0.226**
Cardiac output x SVR		**0.708 (0.139)**	**<0.001**	**0.457**

Data were analysed in multivariate regression models. DNP, diabetic nephropathy; HNP, hypertensive nephropathy; CKD, chronic kidney disease; β, regression coefficient; PO, population origin; ESA, erythropoietin stimulating agents; MAP, mean arterial pressure; SVR, systemic vascular resistance.

[a]Variables that were adjusted for comprised age, female sex, black population origin, exercising status, haemoglobin concentration, erythropoietin stimulating agent use, body mass index and heart rate.

Table S7. Confounder and mutually independent potential impact of DNP and HNP on CFPWV in 83 CKD patients without CVD

Characteristics	Cumulative R^2	β (SE)	*p* value	Std. β
Adjusted variables[a]	0.217			
+HNP	0.221	0.570 (0.968)	0.6	0.078
+DNP	0.272	**2.114 (0.976)**	**0.03**	**0.254**
+HNP and	0.275	-0.612 (1.091)	0.6	1.091
DNP		**2.437 (1.138)**	**0.03**	**0.293**
+HNP and	0.302	-0.563 (1.080)	0.6	-0.077
DNP and		**2.378 (1.127)**	**0.03**	**0.286**
MAP		0.053 (0.035)	0.1	0.181
+HNP and	0.319	-0.667 (1.093)	0.5	-0.091
DNP and		**2.503 (1.118)**	**0.02**	**0.301**
Cardiac output		**0.543 (0.269)**	**0.04**	**0.260**
+HNP and	0.292	-0.636 (1.114)	0.6	-0.086
DNP and		**2.486 (1.140)**	**0.03**	**0.299**
Log SVR		-4.380 (3.359)	0.2	-0.170
+HNP and	0.331	-0.630 (1.092)	0.6	-0.086
DNP and		**2.490 (1.118)**	**0.03**	**0.300**
Cardiac output and		1.089 (0.590)	0.07	0.521
Log SVR		7.520 (7.243)	0.3	0.292
+HNP and	0.299	-0.490 (1.107)	0.7	-0.066
DNP and		**2.386 (1.135)**	**0.04**	**0.287**
Cardiac output x SVR		0.053 (0.035)	0.1	0.182

Data were analysed in multivariate regression models. DNP, diabetic nephropathy; HNP, hypertensive nephropathy; CFPWV, carotid-femoral pulse wave velocity; CKD, chronic kidney disease; β, regression coefficient; PO, population origin; ESA, erythropoietin stimulating agents; MAP, mean arterial pressure; SVR, systemic vascular resistance.
[a]Variables that were adjusted for comprised age, female sex, black population origin, exercising status, haemoglobin concentration, erythropoietin stimulating agent use, body mass index and heart rate.

Table S8. Confounder and mutually independent potential impact of DNP and HNP on CFPWV non-dialysis and dialysis CKD patients

	Non-dialysis CKD patients (n=67)				Dialysis (n=48)			
Characteristics	Cumulative R^2	β (SE)	*p* value	Std. β	Cumulative R^2	β (SE)	*p* value	Std. β
Adjusted variables[a]	0.260				0.236			
+HNP	0.263	-0.466 (1.185)	0.7	-0.064	0.284	2.284 (1.570)	0.1	0.246
+DNP	0.303	2.179 (1.319)	0.1	0.279	0.326	**3.623 (1.758)**	**0.04**	**0.361**[b]
+HNP and	0.324	-1.423 (1.246)	0.2	-0.196	0.328	0.539 (1.975)	0.8	0.133
DNP		2.815 (1.428)	0.05	0.360		3.235 (2.279)	0.1	0.322[c]
+HNP and	0.328	-1.510 (1.270)	0.2	-0.208	0.438	-0.208 (1.861)	0.9	0.038
DNP		3.013 (1.496)	0.05	0.385		3.326 (2.118)	0.1	0.331[d]
MAP		-0.026 (0.054)	0.6	-0.074		**0.120 (0.050)**	**0.02**	**0.374**
+HNP and	0.327	-1.213 (1.373)	0.4	-0.165	0.459	0.013 (1.811)	1.0	0.001
DNP and		2.874 (1.494)	0.06	0.367		3.207 (2.078)	0.1	0.319[e]
Cardiac output		0.237 (0.329)	0.5	0.117		**1.058 (0.392)**	**0.01**	**0.482**
+HNP and	0.326	-1.226 (1.373)	0.4	-0.167	0.379	0.284 (1.935)	0.9	0.031
DNP and		2.979 (1.494)	0.05	0.380		3.451 (2.230)	0.1	0.344[f]
Log SVR		-2.296 (3.351)	0.5	-0.105		-7.770 (4.908)	0.1	-0.291
+HNP and	0.327	-1.211 (1.392)	0.4	-0.165	0.497	-0.051 (1.777)	1.0	-0.006
DNP and		2.892 (1.564)	0.07	0.369		2.790 (2.058)	0.1	0.186[g]
Cardiac output and		0.197 (0.911)	0.8	0.097		**2.112 (0.812)**	**0.01**	**0.963**
Log SVR		-0.436 (9.257)	1.0	-0.020		13.998 (9.501)	0.1	0.525
+HNP and	0.323	-1.560 (1.348)	0.2	-0.212	0.438	-0.208 (1.861)	0.9	-0.022
DNP and		**3.173 (1.561)**	**0.04**	**0.405**		3.326 (2.118)	0.1	0.331[h]
Cardiac output x SVR		-0.030 (0.055)	0.6	-0.083)		**0.120 (0.05)**	**0.02**	**0.374**

Significant associations are given in bold.

[a]Variables that were adjusted for comprised age, female sex, black population origin, exercising status, haemoglobin concentration, erythropoietin stimulating agent use, body mass index and heart rate.

[b]p=0.6 versus the relation with CFPWV in non-dialysis patients.

[c]p=0.8 versus the relation with CFPWV in non-dialysis patients.

[d]p=0.7 versus the relation with CFPWV in non-dialysis patients.

[e]p=0.8 versus the relation with CFPWV in non-dialysis patients.

[f]p=0.8 versus the relation with CFPWV in non-dialysis patients.

[g]p=0.3 versus the relation with CFPWV in non-dialysis patients.

[h]p=0.7 versus the relation with CFPWV in non-dialysis patients.

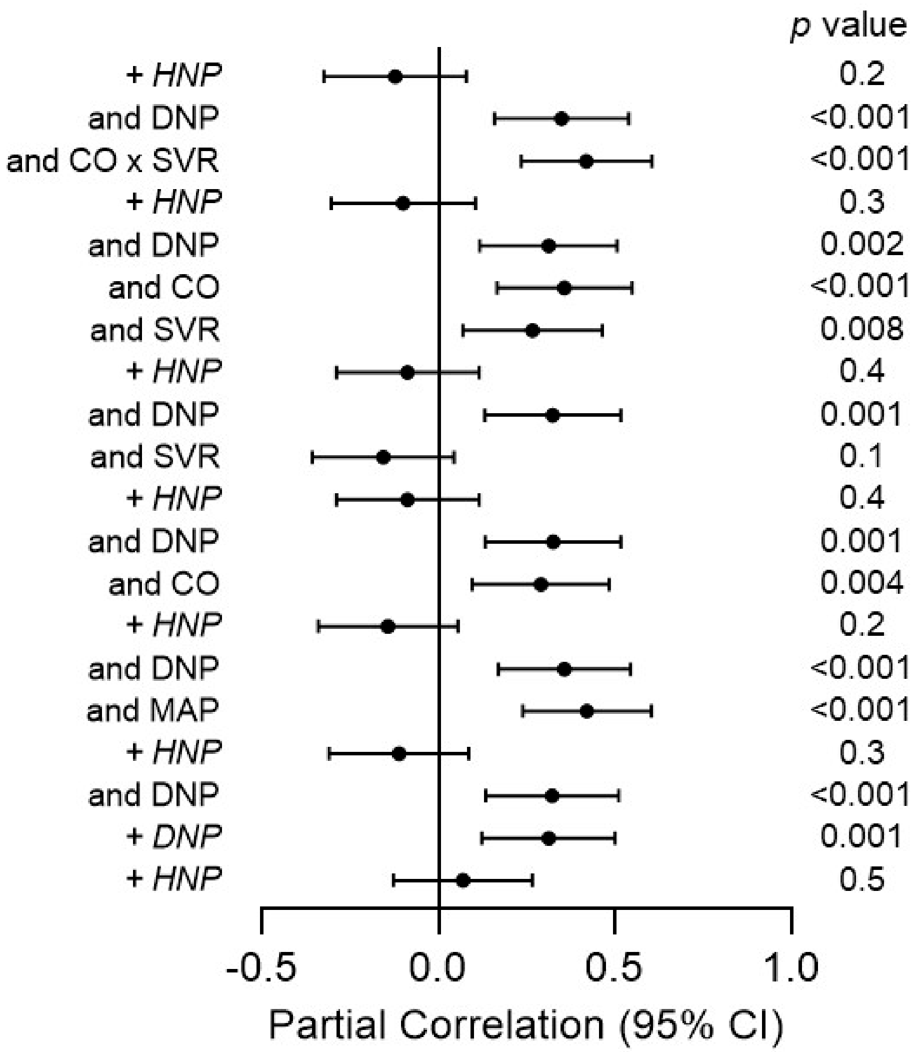

Figure S1. Partial correlations for the models in Table 4 (peripheral pulse pressure). DNP, diabetic nephropathy; HNP, hypertensive nephropathy; MAP; mean arterial pressure; log, logarithmically transformed; SVR, systemic vascular resistance.

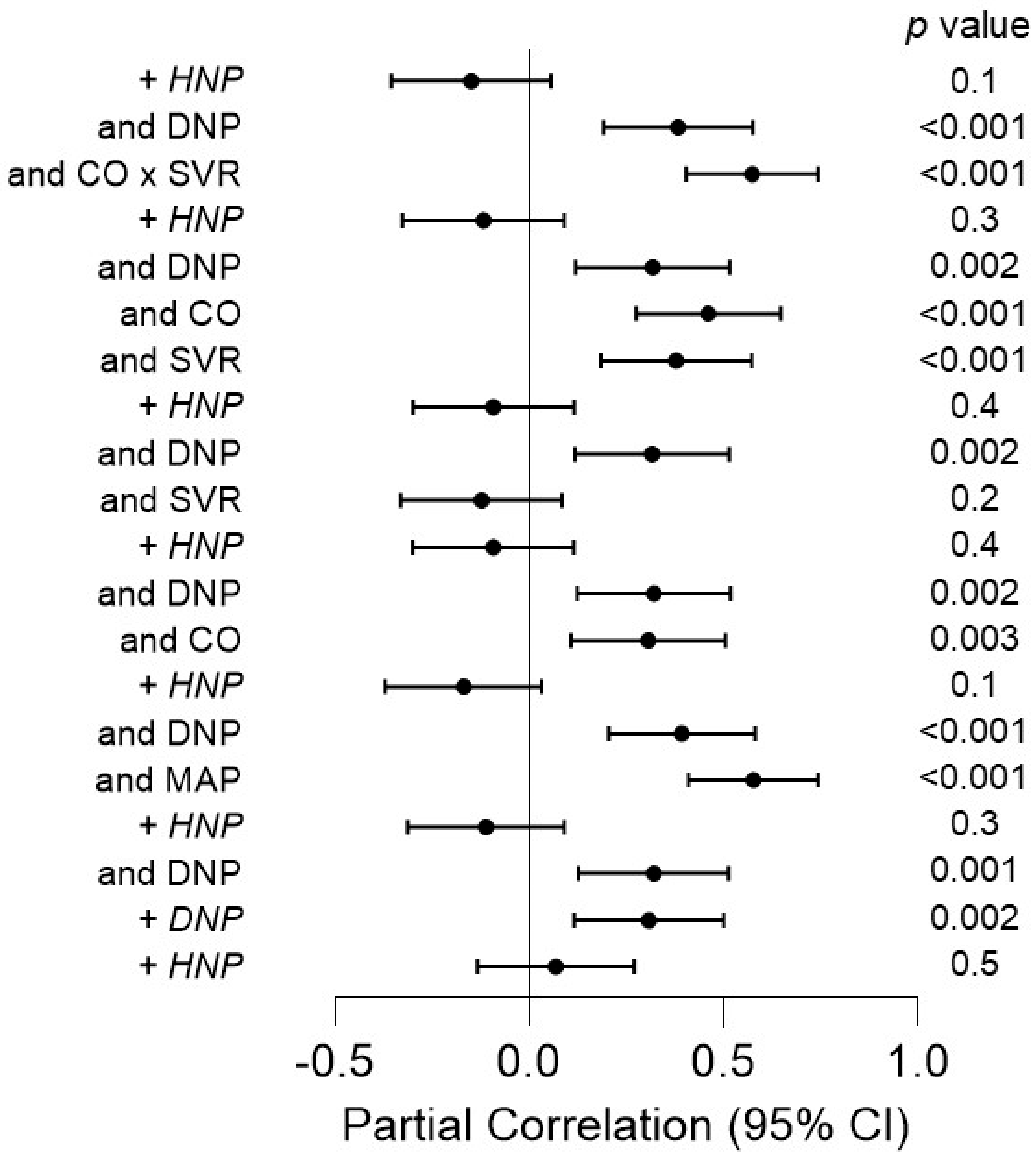

Figure S2. Partial correlations for the models in Table 5 (central pulse pressure). DNP, diabetic nephropathy; HNP, hypertensive nephropathy; MAP; mean arterial pressure; log, logarithmically transformed; SVR, systemic vascular resistance.

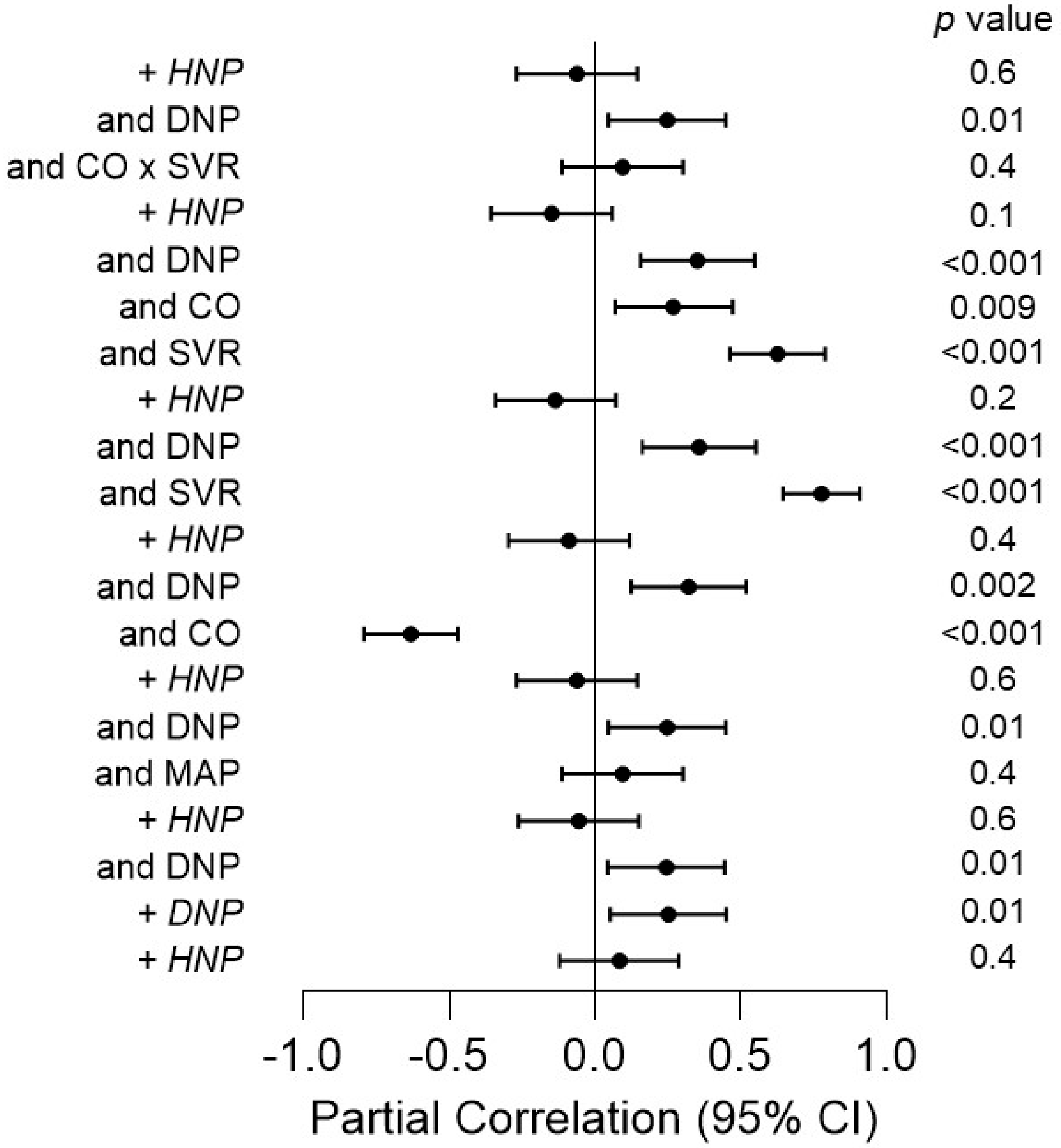

Figure S3. Partial correlations for the models in Table 6 (the inverse of total arterial compliance). DNP, diabetic nephropathy; HNP, hypertensive nephropathy; MAP; mean arterial pressure; log, logarithmically transformed; SVR, systemic vascular resistance.

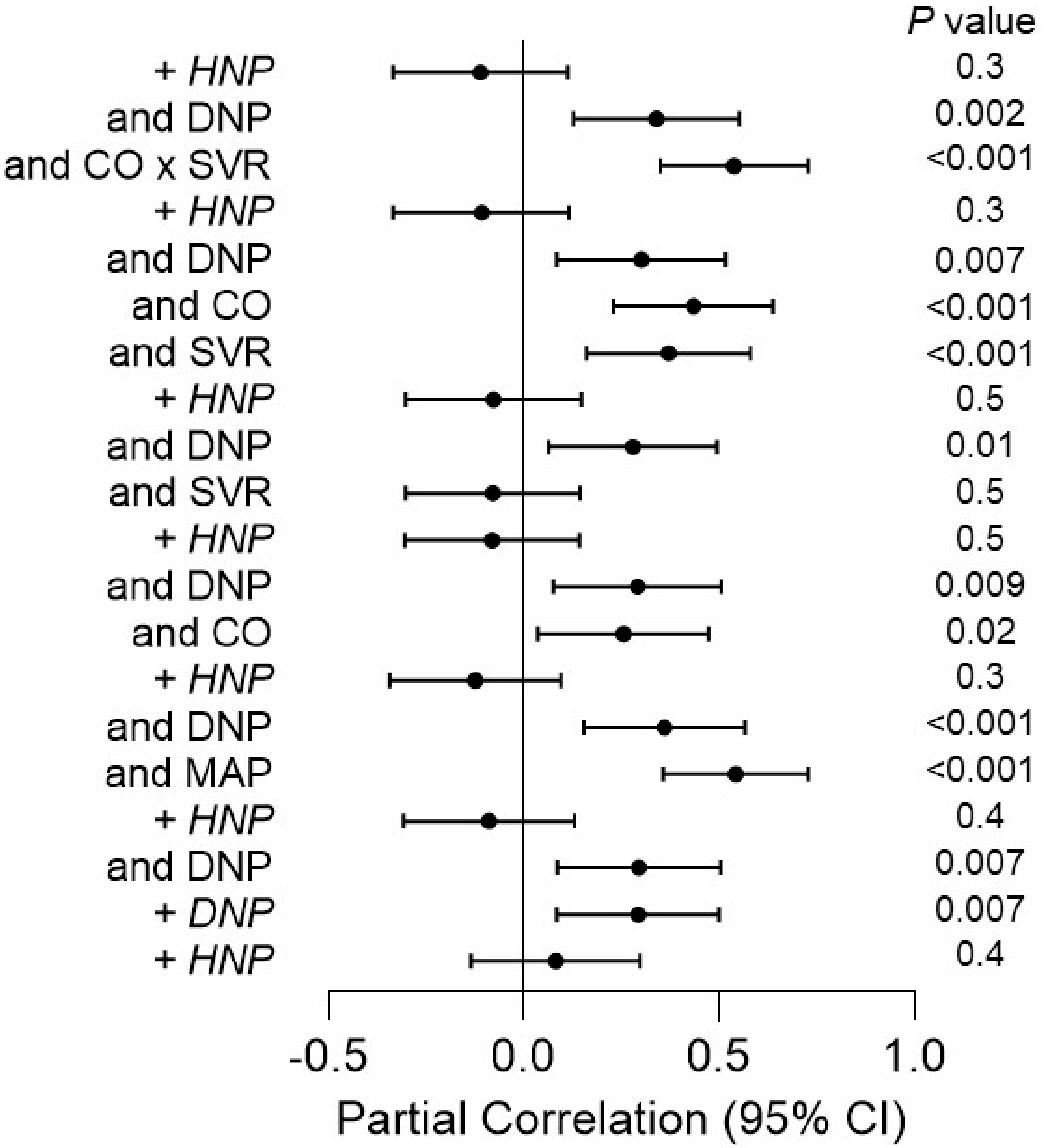

Figure S4. Partial correlations for the models in Table S3 (reflective wave amplitude). DNP, diabetic nephropathy; HNP, hypertensive nephropathy; MAP; mean arterial pressure; log, logarithmically transformed; SVR, systemic vascular resistance.

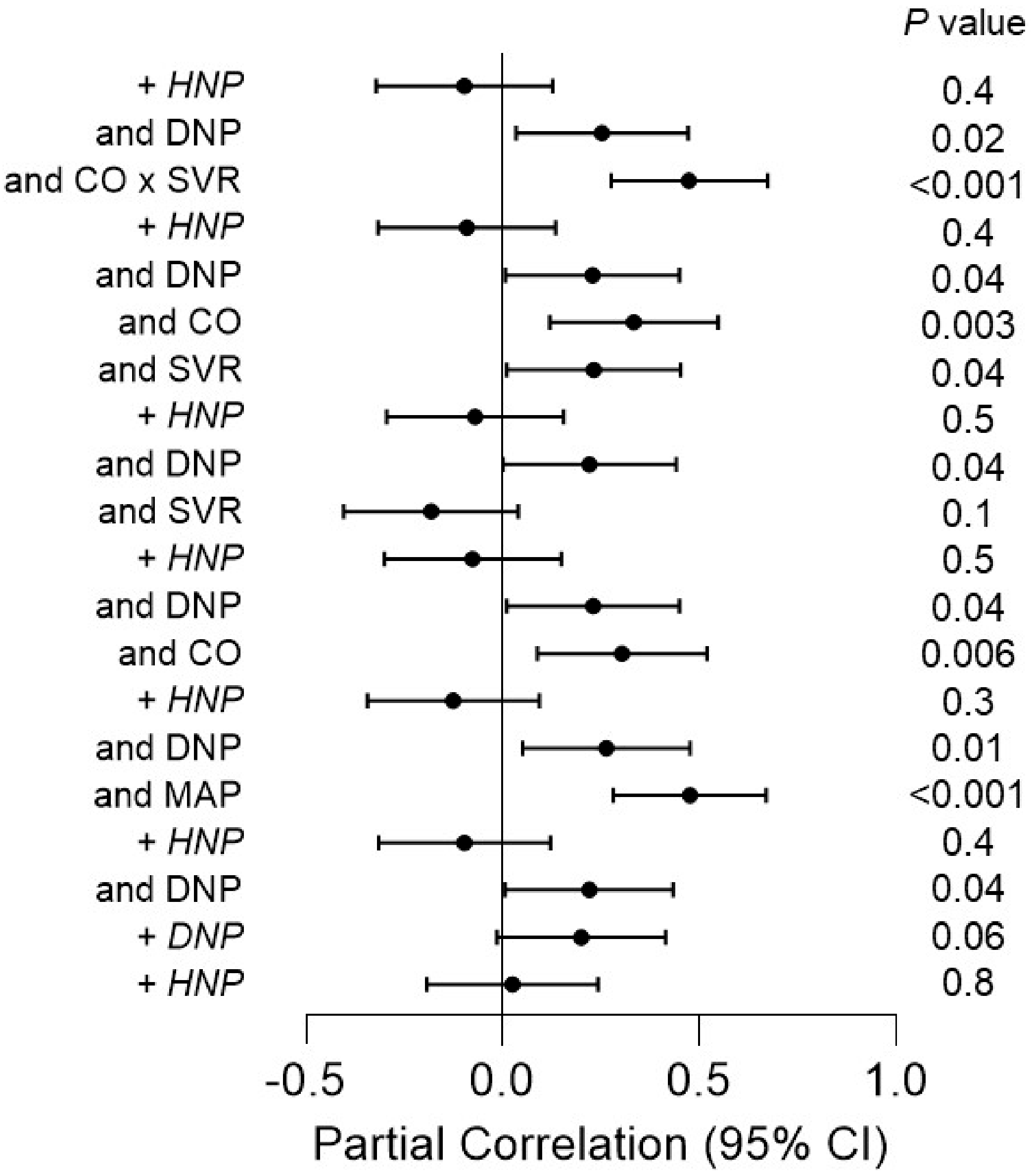

Figure S5. Partial correlations for the models in Table S4 (forward wave amplitude). DNP, diabetic nephropathy; HNP, hypertensive nephropathy; MAP; mean arterial pressure; log, logarithmically transformed; SVR, systemic vascular resistance.
